# 4-Phenylbutyrate ameliorates apoptotic neural cell death in Down syndrome by reducing protein aggregates

**DOI:** 10.1038/s41598-020-70362-x

**Published:** 2020-08-20

**Authors:** Katsuya Hirata, Toshihiko Nambara, Keiji Kawatani, Nobutoshi Nawa, Hidetaka Yoshimatsu, Haruna Kusakabe, Kimihiko Banno, Ken Nishimura, Manami Ohtaka, Mahito Nakanishi, Hidetoshi Taniguchi, Hitomi Arahori, Kazuko Wada, Keiichi Ozono, Yasuji Kitabatake

**Affiliations:** 1 Department of Pediatrics, Graduate School of Medicine, Osaka University, Suita, Osaka 565-0871 Japan; 2 Department of Neonatal Medicine, Osaka Women’s and Children’s Hospital, Izumi, Osaka 594-1101 Japan; 3 Department of Physiology II, Nara Medical University, Kashihara, Nara 634-8521 Japan; 4 Laboratory of Gene Regulation, Faculty of Medicine, University of Tsukuba, Tsukuba, Ibaraki 305-8575 Japan; 5 Biotechnology Research Institute for Drug Discovery, National Institute of Advanced Industrial Science and Technology (AIST), Tsukuba, Ibaraki 305-8562 Japan; 6 TOKIWA-Bio, Inc, Tsukuba Center Inc. (TCI), Tsukuba, Ibaraki 305-0047 Japan

**Keywords:** Cell biology, Neuroscience

## Abstract

Individuals with Down syndrome (DS) commonly show unique pathological phenotypes throughout their life span. Besides the specific effects of dosage-sensitive genes on chromosome 21, recent studies have demonstrated that the gain of a chromosome exerts an adverse impact on cell physiology, regardless of the karyotype. Although dysregulated transcription and perturbed protein homeostasis are observed in common in human fibroblasts with trisomy 21, 18, and 13, whether and how this aneuploidy-associated stress acts on other cell lineages and affects the pathophysiology are unknown. Here, we investigated cellular stress responses in human trisomy 21 and 13 neurons differentiated from patient-derived induced pluripotent stem cells. Neurons of both trisomies showed increased vulnerability to apoptotic cell death, accompanied by dysregulated protein homeostasis and upregulation of the endoplasmic reticulum stress pathway. In addition, misfolded protein aggregates, comprising various types of neurodegenerative disease-related proteins, were abnormally accumulated in trisomic neurons. Intriguingly, treatment with sodium 4-phenylbutyrate, a chemical chaperone, successfully decreased the formation of protein aggregates and prevented the progression of cell apoptosis in trisomic neurons. These results suggest that aneuploidy-associated stress might be a therapeutic target for the neurodegenerative phenotypes in DS.

## Introduction

Down syndrome (DS) is the most common genetic chromosomal disorder, affecting approximately one in 700–800 newborns^1^. All individuals with DS exhibit cognitive and learning deficits, with broad variation in phenotypes and severities^2^. Studies using in vitro and in vivo experiments with postmortem brains, neural stem/progenitor cells (NPCs), or induced pluripotent stem cells (iPSCs) derived from DS patients and DS mouse models have shown various levels of neuropathology, including reduced neurogenesis, impaired neuronal maturation, and accelerated neural cell death. These features arise from the extra copy of human chromosome 21 (Hsa21), and specific DS phenotypes are generally thought to be derived from the increased expression of a specific subset of dosage-sensitive genes on Hsa21^3^. Based on this ‘gene dosage imbalance’ hypothesis, multiple studies have been conducted to identify the genes responsible for impaired neural development^4–6^. Human neural progenitors isolated from foetal brains with DS show vulnerability to oxidative stress and preference for gliogenesis, which is accompanied by elevated expression levels of *S100B* and *APP* genes^7^. The *DYRK1A* gene is reported to affect the differentiation of mouse cortical NPCs and play a critical role in brain morphogenesis^8^. The administration of a DYRK1A inhibitor was shown to rescue proliferative deficits in NPCs derived from DS model mice^9^; thus, these dosage-sensitive genes are potential therapeutic targets in DS.

In addition to congenital intellectual disabilities that originate from foetal stages and develop during childhood, adults with DS are at a markedly increased risk of dementia^10^. Cumulative neuroimaging and autopsy studies of DS patients showed typical pathological manifestation of the early onset of Alzheimer disease (AD), which is consistent with the direct result of *APP* gene-dosage effects. However, an increasing number of clinical case reports demonstrate that some adolescents and young adults with DS show more rapid and atypical deterioration in cognitive function^11^. Clinical onset of this regression is sudden, and the evolution is quite variable. This acute type of cognitive regression in DS cannot be explained by the simple gene-dosage effects on APP processing, and detailed mechanisms for these modifications in dementia have not been elucidated yet.

Besides the direct chromosome-specific effects, recent studies provide evidence that aneuploidy exerts common adverse effects on cell physiology^12–14^. Aneuploid cells of yeasts, plants, mice, and humans have been found to exhibit severe proliferation defects, metabolic alterations, or genome instability, regardless of the origin of the aneuploid chromosome^15^. While RNA expression from aneuploid autosomes is largely proportional to their copy number, transcriptional levels of genes on other chromosomes are also affected, causing the deregulation of global gene transcription^16^. Unbalanced transcription causes a mismatch between protein production, degradation, and accumulation of misfolded proteins, resulting in disrupted protein homeostasis^17^. We previously reported that dermal fibroblasts derived from patients with trisomy 21, 18, and 13 showed a severe impairment of cell proliferation and enhanced premature senescence^18^. These common pathological features coincide with the dysregulation of global gene transcription and perturbed protein homeostasis, leading to the excessive accumulation of protein aggregates. We found that treatment with sodium 4-phenylbutyrate (4-PBA), a potent chemical chaperone compound, successfully reduced the protein aggregation in trisomic fibroblasts and prevented the progression of premature senescence in secondary fibroblasts derived from trisomy 21 iPSCs. To elucidate whether and how aneuploidy-associated stress affects neural cell lineages, and to determine the possibilities of its role as a therapeutic target for neural dysfunctions in DS, we examined cellular stress responses in human trisomic neurons differentiated from disease-specific iPSCs. Furthermore, we explored whether 4-PBA can alleviate the disruption in protein homeostasis, which causes pathological phenotypes in trisomic neurons.

## Results

### Generation of trisomy-specific iPSCs and neural differentiation

**Figure 1:**
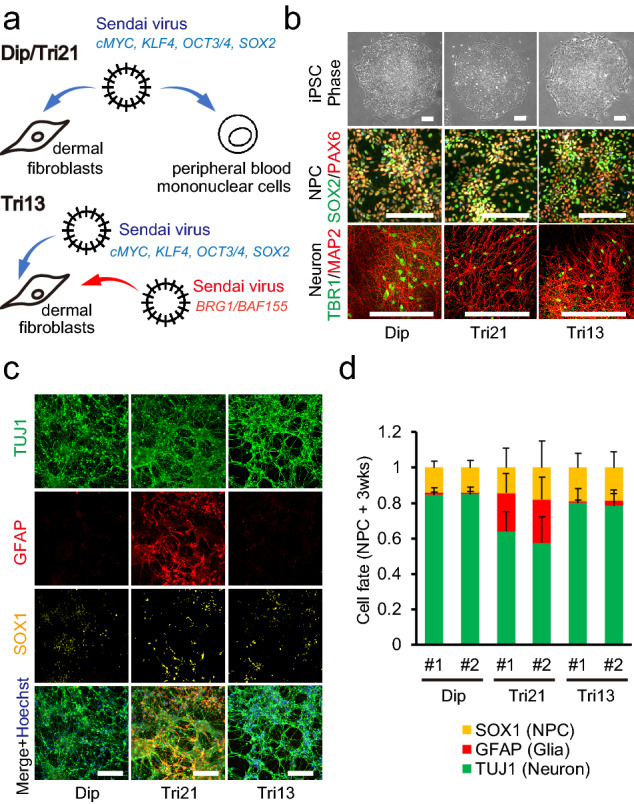
Establishment of trisomy-specific iPSCs and early enhanced gliogenesis in trisomy 21 NPCs. (**a**) Schematic diagram showing the reprogramming processes of diploid control, trisomy 21, and trisomy 13 iPSCs. (**b**) iPSCs generated from diploid, trisomy 21, and trisomy 13 patients showed ESC-like morphology (phase image). NPCs differentiated from iPSCs expressed the NPC markers SOX2 and PAX6. Neurons differentiated from NPCs after 3 months expressed the pan-neural marker MAP2 and cortical neural marker TBR1. Scale bar = 200 μm. (**c**) Representative image of immunocytochemistry for a neural marker (TUJ1), glial marker (GFAP), and NPC marker (SOX1) after 3 weeks of differentiation from NPCs. Scale bar = 200 μm. (**d**) Quantification of the ratio of each marker-positive cell/Hoechst 33342-positive cell after 3 weeks of differentiation from NPCs. Data are presented as mean ± SEM. n = 3 per clone.

To examine whether aneuploidy-associated stresses can affect cellular physiology and be involved in pathological mechanisms in the central nervous system, we generated disease-specific iPSC lines from patients with trisomies 13 and 21 and age-matched healthy individuals. Reprogramming factors with or without the chromatin-remodelling complex *BRG1/BAF155* were transduced into dermal fibroblasts and peripheral blood mononuclear cells using Sendai virus to obtain iPSCs with trisomy 21 (two lines, three clones; Tri21), trisomy 13 (one line, two clones; Tri13), and diploid status (two lines, two clones; Dip) (Fig. 1a, Patients’ clinical manifestations are listed in Table S1)^19,20^. Karyotype analysis of iPSCs exhibited no acquired abnormalities and confirmed normal diploidy, trisomy 21, or trisomy 13 in the appropriate lines, except for the presence of a pericentric inversion of chromosome 3 in the Tri13#2 line (Fig. S1a). All iPSC clones used in the present study showed the typical morphology and expression of pluripotent markers, including OCT3/4 and SSEA4 (Fig. S1b). The generated iPSCs could form teratomas that showed structures corresponding to three germ layers (Fig. S1c).

To examine whether aneuploidy-associated stress affects the process of neural development, these iPSCs were differentiated into a neural lineage by generating embryoid bodies (EBs) using published protocols with some modifications^21^ (Fig. 1b, Fig. S1b and d). Diploid- and trisomy-iPSC-derived NPCs exhibited homogeneous morphology, forming typical rosette-like patterns and equally expressed the NPC markers SOX1, SOX2, PAX6, or Nestin (Fig. 1b, Fig. S1b). These NPCs were efficiently induced to generate TBR1-positive cortical neurons, with positive ratios of 62%, 64%, and 58% in the Dip, Tri21, and Tri13 lines, respectively (Fig. 1b). Differentiation efficiencies of diploid and trisomic NPCs were evaluated by immunostaining after 3 weeks of neural induction. There was no significant difference in the population of SOX1-positive NPCs, GFAP-positive astrocytes, and TUJ1-positive neurons between Dip- and Tri13-NPC-derived cells (Fig. 1c,d). In contrast, the proportion of GFAP-positive cells was significantly higher in the Tri21 line compared with that in the Dip or Tri13 lines, suggesting that this phenomenon is specific to trisomy 21.

### Protein homeostasis was perturbed in trisomic NPCs

**Figure 2:**
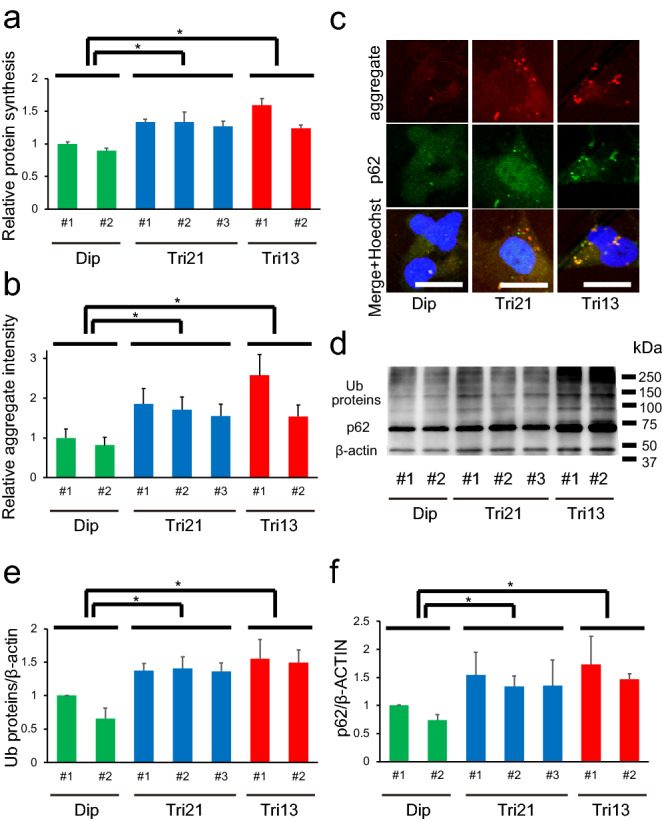
Perturbed protein homeostasis in human iPSC-derived NPCs in trisomy syndromes. (**a**) Relative protein synthesis per cell in NPCs. Data are presented as mean ± SEM normalized to the average of Dip #1 clone. *P < 0.05, n = 4–6 per clone. (**b**) Relative aggregate intensity per cell in NPCs. Data are presented as mean ± SEM normalized to the average of Dip #1 clone. *P < 0.05, n = 4–6 per clone. (**c**) Representative images showing immunocytochemical staining for aggregates (red); p62 (green); and merged images of aggregates, p62, and Hoechst 33342 in NPCs. Scale bar = 20 μm. (**d**) Western blot of ubiquitinylated (Ub) proteins and p62 in NPCs, using anti-Ub-protein and anti-p62 antibodies. Full immunoblot images of them are shown in Figure S2. (**e**, **f**) Quantification of the western blot bands of the Ub proteins (**e**) and p62 (**f**). β-Actin, loading control. Data are presented as mean ± SEM normalized to the average of Dip #1 clone. *P < 0.05, n = 4 per clone.

To examine whether aneuploidy-associated proteotoxic stress can affect cellular function in the neural lineage, protein synthesis of NPCs was evaluated using an amino acid analogue of methionine. Rates of newly synthesized proteins were significantly elevated in trisomy 21- and trisomy 13-derived NPCs compared with that in diploid NPCs (Fig. 2a). In addition, cellular protein aggregates detected by a molecular rotor dye^22^ were significantly increased in trisomic NPC lines, similar to trisomic human fibroblasts (Fig. 2b and c).

Cellular proteostasis is generally maintained by two proteolytic systems, the ubiquitin–proteasome system and autophagy^23^. When misfolded proteins are produced in excess and cellular proteostasis machinery is overburdened with aggregation-prone proteins in response to cellular stresses, protein aggregates are concentrated to form aggresomes. Trisomy 21 and trisomy 13 NPCs contained excessive amounts of high-molecular-weight ubiquitinated proteins (Fig. 2d,e, Fig. S2) and the ubiquitin-binding protein p62 (Fig. 2d,f). Moreover, these aggresomes were colocalized with p62 proteins (Fig. 2c). These results are consistent with those of studies reporting elevated levels of poly-ubiquitinated proteins in the postmortem brains of patients with DS and proteostasis dysfunction in DS cells^24–26^, and suggest that protein degradation machinery increases in response to trisomy-induced proteotoxic changes.

### Neural differentiation of trisomy iPSCs via forced expression of NGN2

**Figure 3:**
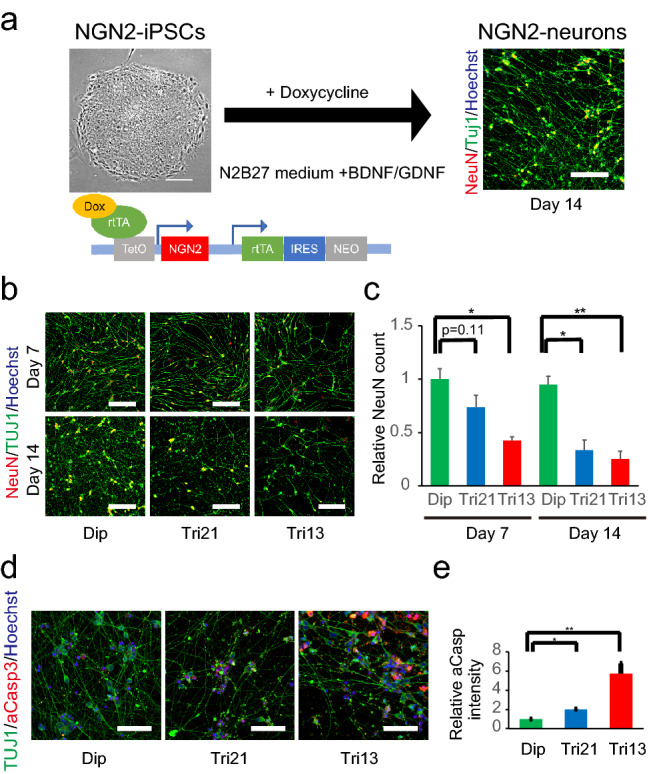
Neurogenesis with Ngn2-inducible iPSCs and decreased cell survival in trisomic neurons. (**a**) NGN2 under the control of the tetracycline operator was introduced into iPSCs using the *piggyBac* vector. Neurons were generated from iPSCs within 14 days. Representative images of neurons from Dip-NGN2-iPSCs and the construct of the NGN2 inducible *piggyBac* vector are shown. Scale bar = 200 μm. (**b**) Representative images showing immunocytochemical staining of TUJ1 and NeuN on days 7 and 14 in diploid control and trisomy neurons. Scale bar = 200 μm. (**c**) Quantitative data of the NeuN-positive cells on days 7 and 14. Data are presented as mean ± SEM normalized to the average of diploid clones on day 7. *P < 0.05, n = 4–6 per clone. (**d**) Representative images showing immunocytochemical staining for cleaved caspase 3 (red), TUJ1 (green), and Hoechst 33342 (blue) in NGN2 neurons on day 14. Scale bar = 100 μm. (**e**) Quantification of relative cleaved caspase 3 intensity in NGN2 neurons on day 14. Data are presented as mean ± SEM normalized to the average of diploid clones. *P < 0.05, **P < 0.001, n = 4 per clone.

We next aimed to evaluate the proteotoxic effect observed in trisomic NPCs in mature neurons. The conventional neural differentiation protocol from human iPSCs generally requires months of tissue culture and renders large-scale analysis difficult. Moreover, the resulting differentiated neurons are often vulnerable and contain varying ratios of glial cells. To robustly generate homogenous neurons, we introduced a transcription factor, neurogenin 2 (*NGN2*), into diploid, trisomy 21, and trisomy 13 iPSCs using the *piggyBac* vector with a tetracycline-inducible gene expression system (NGN2-iPSCs), followed by drug selection for stable-line establishment (Fig. 3a)^27,28^. NGN2-iPSCs maintained undifferentiated morphology and expressed high levels of the pluripotent markers OCT3/4 and NANOG (Fig. S3). The administration of doxycycline and forced expression of NGN2 effectively converted NGN2-iPSCs into neuron-like cells and produced apparently mature neuronal morphology on day 14 (NGN2-neurons) (Fig. S4). NGN2-neurons expressed significantly increased levels of NGN2, as well as increased levels of the neural markers MAP2, TUJ1, VGLUT1, and BRN2. In addition, the expression of pluripotent markers was abolished in NGN2-neurons, consistent with the efficient conversion of iPSCs into neurons (Fig. S3). There were no differences in the differentiation propensities between diploid and trisomic NGN2-neurons.

### Impaired cell viability and elevated apoptotic cell death in trisomic neurons

Evaluation of cell proliferation using the thymidine analogue EdU (5-ethynyl-2′-deoxyuridine) revealed that the proportion of proliferative cells was around 10% on day 7, whereas these signals were rarely detected on day 14, indicating a rapid loss of proliferative properties by the forced expression of NGN2 (Fig. S5). There were no significant differences in the proliferation rate between diploid and trisomic neurons. Although NeuN-positive neuronal cell numbers seemed to be unchanged between day 7 and day 14 in diploid cells, there was a significant decrease in the number of neurons in both trisomy 21 and trisomy 13 (Fig. 3b,c). Assessment of cell apoptosis by immunohistochemistry for cleaved caspase 3 showed that the fluorescence intensity of apoptotic cells was significantly higher in Tri21- and Tri13-NGN2-neurons than that of diploid NGN2-neurons (Fig. 3d,e). To exclude the possible effects of genetic variability, this result was validated in another experiment using isogenic control cell lines: a corrected disomy 21 (cDi21) iPSC line, in which a single copy of chromosome 21 was removed from a Tri21 iPSC^29^, and a rescued disomy 13 (rDi13) iPSC line, in which a copy of chromosome 13 was spontaneously eliminated from a Tri13 iPSC line by trisomy rescue (Fig. S6a). Cleaved caspase 3-positive apoptotic cells in NGN2-transduced neurons were significantly increased in Tri21- and Tri13-NGN2-neurons compared to in cDi21- and rDi13-NGN2-neurons, respectively (Fig. S6b). These results indicated that trisomies 21 and 13 caused apoptotic cell death in neurons, which led to the decrease in the neural population.

### Abnormally high accumulation of protein aggregates containing non-specific proteins in trisomic neurons

**Figure 4:**
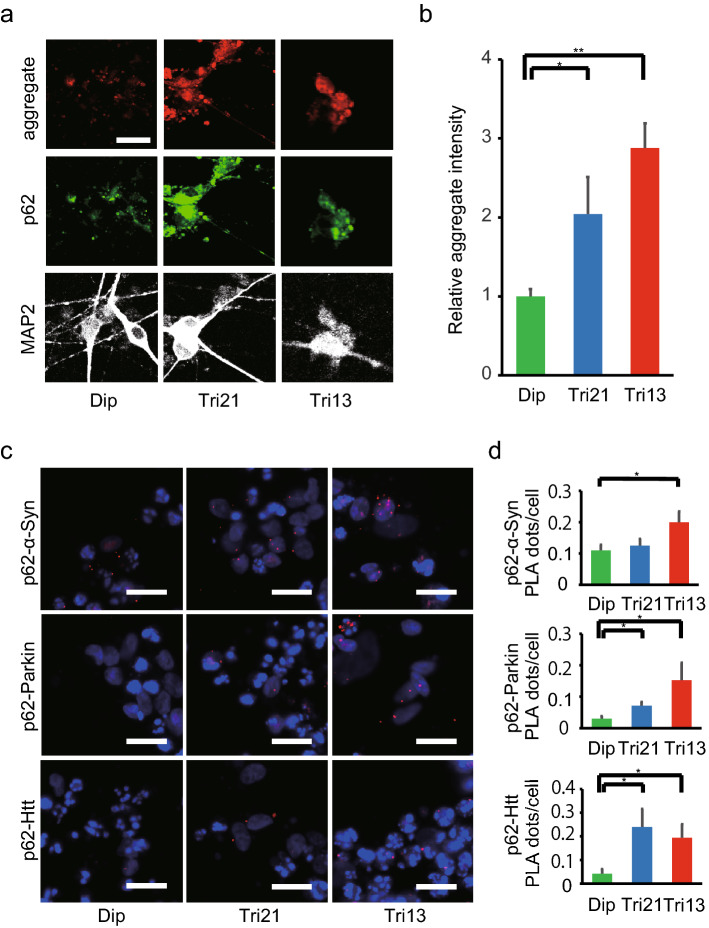
Aggregation of neurodegenerative disorder-associated proteins in trisomic neurons. (**a**) Representative images showing immunocytochemical staining for aggregates (red), p62 (green), and MAP2 (white) in NGN2-neurons on day 14. Scale bar = 20 μm. (**b**) Relative aggregate intensity per cell in NGN2-neurons (D14). *P < 0.05, **P < 0.001, n = 5–13 per clone. (**c**) PLA to detect the interaction between p62 and α-Syn, Parkin, or Htt in NGN2-neurons. PLA signals are seen as red dots. Hoechst 33342 counterstaining appears in blue. Scale bar = 20 μm. (**d**) Quantification of PLA signals (red dots) per cell. Data are presented as mean ± SEM. *P < 0.05, n = 3–6 per clone.

We next evaluated the accumulation of protein aggregates in NGN2-neurons and found that aggregates containing p62 were significantly increased in trisomic neurons compared with diploid neurons (Fig. 4a,b) or isogenic cDi21/rDi13 neurons (Fig. S6c), consistent with the results of trisomic NPCs (Fig. 2c,d). The accumulation of misfolded proteins containing p62 in proteinaceous intracellular inclusions is a prominent pathological feature common to many age-related neurodegenerative diseases, including Parkinson disease (PD), Alzheimer disease (AD), and Huntington disease (HD)^30,31^. These disorders show aberrant aggregation of specific proteins such as α-Synuclein (α-Syn), Parkin, and Huntingtin (Htt)^32^; therefore, we sought to determine which kinds of proteins mainly form these aggregates in trisomic neurons. To examine direct interactions between p62 and these neurodegenerative disease-associated proteins in situ, the proximity ligation assay (PLA), an antibody-based technique to study protein–protein interaction, was performed on day 14 NGN2-neurons^22^. Interestingly, PLA signals of p62 and α-Syn, Parkin, or Htt were significantly higher in trisomic neurons compared with those of diploid neurons (Fig. 4c,d). Dermal fibroblasts derived from patients with trisomies exhibited global transcriptional amplification^18^, and studies of aneuploid yeast revealed novel aneuploidy-associated gene expression signatures^33^. These results suggest that protein aggregates observed in neurons with trisomies may be formed by the accumulation of various types of misfolded proteins instead of specific proteins.

### 4-Phenylbutyric acid ameliorated cellular stress and neuronal loss in trisomic neurons

**Figure 5:**
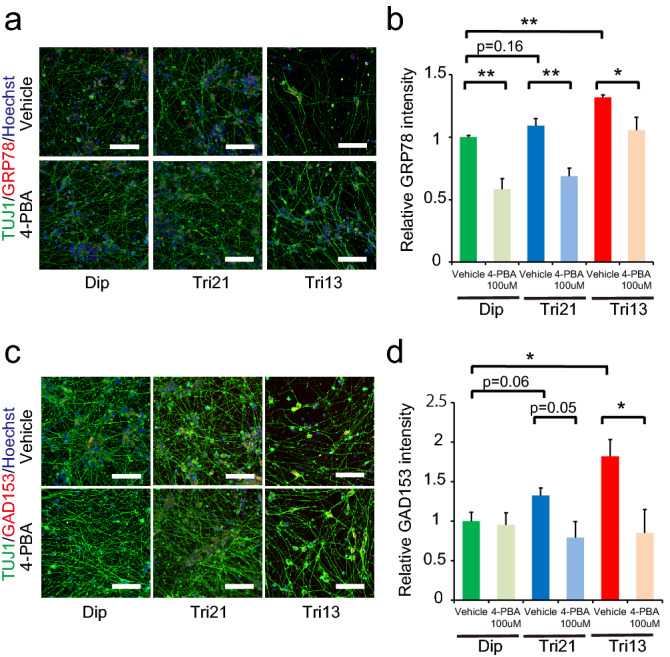
Elevated ER stress in trisomic neurons, which was ameliorated by 4-phenylbutyric acid. (**a**) Representative images showing immunocytochemical staining for GRP78 (BIP) (red) and TUJ1 (green) in diploid and trisomic NGN2-neurons treated with vehicle or 4-PBA. Hoechst 33342 (blue), counterstaining. Scale bar = 200 μm. (**b**) Quantification of the relative intensity of GRP78 (BIP) in NGN2-neurons treated with vehicle or 4-PBA. Data are presented as mean ± SEM normalized to the average of diploid clones. *P < 0.05, n = 4 per clone. (**c**) Representative images showing immunocytochemical staining for GAD154 (CHOP) (red) and TUJ1 (green) in diploid and trisomic NGN2-neurons treated with vehicle or 4-PBA. Hoechst 33342 (blue), counterstaining. Scale bar = 200 μm. (**d**) Quantification of the relative intensity of GAD153 (CHOP) in NGN2-neurons treated with vehicle or 4-PBA. Data are presented as mean ± SEM normalized to the average of diploid clones. *P < 0.05, n = 4 per clone.

The accumulation of aberrant proteins disrupts endoplasmic reticulum (ER) quality control pathways, which can eventually trigger neuronal cell death^34^. To evaluate whether ER function was perturbed in trisomic neurons, we assessed the expression of ER stress marker proteins by immunofluorescence analysis. Signal intensities of GRP78/BIP, a central regulator for ER stress, were significantly higher in trisomic neurons than in those of diploid neurons (Fig. 5a,b). Moreover, the expression of GAD153/CHOP, one of the components of the ER stress-induced apoptosis pathway, was similarly upregulated in trisomic neurons (Fig. 5c,d), suggesting that accelerated ER stress in trisomy causes cellular apoptosis in neurons.

**Figure 6:**
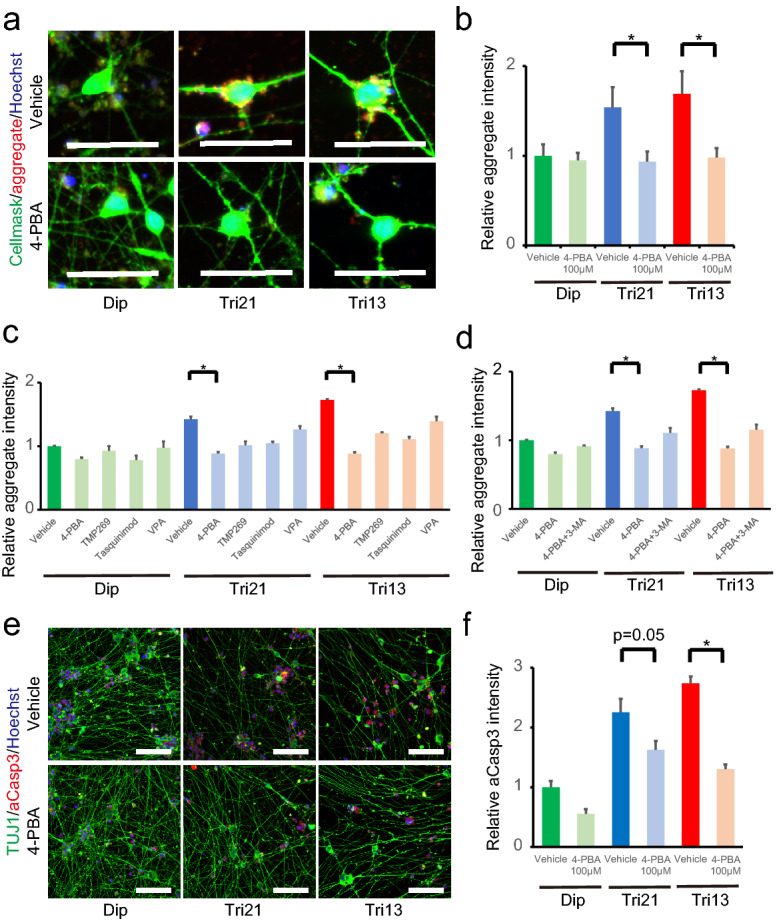
4-PBA ameliorated aggregation and neuronal loss in trisomic neurons. (**a**) Representative images showing immunocytochemical staining for aggregates (red) in diploid and trisomic NGN2-neurons treated with vehicle or 4-PBA. CellMask (green) and Hoechst 33342 (blue), counterstaining. Scale bar = 50 μm. (**b**) Quantification of relative aggregate intensity per cell in NGN2-neurons on day 14. Data are presented as mean ± SEM normalized to the average of diploid clones. *P < 0.05, n = 5–7 per clone. (**c**) Quantification of relative aggregate intensity per cell in diploid and trisomic NGN2-neurons treated with vehicle or HDAC inhibitors on day 14. Data are presented as the mean ± SEM normalised to the average of diploid clones treated with vehicle. *P < 0.05, n = 3–6 per clone. (**d**) Quantification of relative aggregate intensity per cell in diploid and trisomic NGN2-neurons treated with vehicle, or 4-PBA with or without 3-MA. Data are presented as the mean ± SEM normalised to the average of diploid clones treated with vehicle. *P < 0.05, n = 3–6 per clone. (**e**) Representative images showing immunocytochemical staining of TUJ1 and cleaved caspase 3 in diploid and trisomic NGN2-neurons treated with vehicle or 4-PBA. Scale bar = 200 μm. (**f**) Quantification of relative cleaved caspase 3 intensity in NGN2-neurons treated with vehicle or 4-PBA. Data are presented as mean ± SEM normalized to the average of diploid clones. *P < 0.05, n = 10–13 per clone. 4-PBA, sodium 4-phenylbutyrate; VPA, valproic acid; 3-MA, 3-methyladenine.

We previously demonstrated that treatment with the chemical compound 4-PBA, which functions as a chemical chaperon and class I and class IIa histone deacetylase (HDAC) inhibitor, effectively reduced the accumulation of protein aggregates in trisomic human fibroblasts and suppressed the progression of premature senescence in secondary fibroblasts derived from human trisomy 21 iPSCs^18^. To explore whether this compound can alleviate the perturbed protein homeostasis causing pathological phenotypes in trisomic neurons, NGN2-iPSCs were treated with 4-PBA upon neural induction. Administration of 4-PBA significantly decreased protein aggregates in both Tri21- and Tri13-NGN2-neurons, with the number of aggregates returning to the same levels as those in diploid neurons (Fig. 6a,b). The signal intensities of GRP78/BIP were significantly decreased in 4-PBA-treated neurons, suggesting the effective reduction of the ER stress response (Fig. 5a,b). Although treatment with valproic acid, another pan-class I/II HDAC inhibitor^35^, or TMP-269 and tasquinimod, selective class IIa HDAC inhibitors^36,37^, showed similar tendencies in reducing protein aggregates, they were less effective, and the reductions were not significant compared with that observed following 4-PBA treatment (Fig. 6c). Moreover, autophagy inhibition by 3-methyladenine (3-MA) in 4-PBA-treated cells partly abolished the suppressive action towards protein aggregation (Fig. 6d). Notably, the intensity ratio of GAD153/CHOP was suppressed in trisomic neurons (Fig. 5c,d), and apoptosis of cleaved caspase 3-positive cells was successfully prevented in Tri21- and Tri13-NGN2-neurons (Fig. 6e,f). These results indicated that 4-PBA might be useful for the prevention of neurodegenerative phenotypes in trisomy syndromes.

## Discussion

Dermal fibroblasts derived from patients with trisomies 21, 18, and 13, which are the only autosomal trisomies resulting in live births, share same pathological features. In the present study, we investigated pathological alterations in DS neurons caused by chromosomal numerical abnormalities and explored its potential as a therapeutic target. To clarify the influence of aneuploidy on these neurons, we generated human trisomy 21- and trisomy 13-specific iPSCs and differentiated them into NPCs and neurons. Compared with trisomy 21, the life expectancy of which has reached 60 years, trisomy 13 (Edwards syndrome) shows more severe phenotypes and often results in stillbirth or early death of the infant^38^. Due to the severe defects in cell proliferation and enhanced premature senescence, the generation of trisomy 13-specific iPSC lines from human dermal fibroblasts was challenging. By introducing the chromatin-remodelling complex *BRG1/BAF155*, together with Yamanaka factors, into the primary fibroblasts, we successfully established trisomy 13 iPSC clones^19,20^.

The brain sizes of foetuses, children, and adults with DS are about 17–20% smaller than healthy control brains^39^, and these anatomical abnormalities are accompanied by the histological alteration of the reduced number of neurons, relatively higher percentage of astrocytes, and increased incidence of apoptotic cell death^40^. Increased GFAP-positive cell ratios in the Tri21-iPSC line observed in our study were consistent with the findings in postmortem tissue from DS patients^41^, a Ts1Cje mouse model of DS^42^, and several in vitro studies^43,44^. These results suggested that iPSCs established in the present study can recapitulate efficient differentiation into the neural lineage and were suitable for evaluating early stages of neural development. Notably, enhanced gliogenesis was observed only in the Tri21 line but not in the Tri13 line, suggesting that this process was exclusively regulated by a specific subset of dosage-sensitive genes located on chromosome 21, including *DYRK1A*, *s100B*, and *APP*, as previously reported^7,44^.

Meanwhile, both enhanced accumulation of protein aggregates and significant increase in apoptotic cell death in trisomy 21 neurons were similarly observed in trisomy 13 iPSC-derived neurons. Unbalanced karyotypes in yeast and mouse cells have been reported to cause proteotoxic stress, triggered by an increase in misfolded proteins^15,45,46^. Our previous study demonstrated that human primary fibroblasts derived from patients with trisomies 21, 18, and 13 contain excess amounts of total RNA and protein, leading to an increase in protein aggregates^18^. Although our study lacks data of trisomy 18 iPSC lines, the same phenotypes have been seen in fibroblast experiments, suggesting that aneuploidy-associated stress may exert common detrimental effects on cellular homeostasis in neurons.

Aggregation of misfolded and insoluble proteins is one of the features most relevant to neurodegenerative diseases. Protein homeostasis, which is generally maintained by fine-tuned control of protein synthesis, folding, and degradation, are disturbed by stress conditions in several pathological diseases, leading to protein misfolding^47^. Increasing evidence has suggested that neurodegenerative diseases are triggered by the deposition of specific proteins, such as Aβ in AD, α-Syn in PD, Htt in HD, and prions in prion diseases. However, this study and our earlier study showed increased accumulation of protein aggregates in fibroblasts with trisomies 13, 18, and 21, and iPSC-derived neurons with trisomies 13 and 21^18^. Because the gene sets located on chromosomes 21, 18, and 13 are different, there are no common combinations of the genes between these trisomies that are attributable to protein misfolding. A protein–protein interaction assay proved that all the signals of α-Syn, Parkin, and Htt rather than specific, single proteins were uniformly increased in the aggregates. Although the mechanism causing protein aggregation in trisomies are not clear, considering that trisomic fibroblasts exhibit global transcriptional amplification^18^, a gene dosage-dependent increase in the expression of trisomic genes on each chromosome may stimulate the broader transcriptional network, leading to the overproduction of various proteins and aggregate formation.

Disruption of the proteostasis network in aneuploidy, including the unfolded protein response, chaperone system, and proteasomal degradation, has been reported in several studies^26,33^. Any failure of proper folding or escape from quality control gives rise to cell malfunctioning, gradually being toxic to the cell physiology, as seen in premature senescence in trisomic fibroblasts, and apoptotic cell death in trisomic neurons^48^. There seems to be a certain correlation between the threshold for the aggregation in vitro and the threshold for disease in humans^49^, and this process has been in focus as a potent therapeutic target. We previously reported that 4-PBA, a chemical chaperone compound, reduced the accumulation of protein aggregates in trisomic fibroblasts. Here, we could confirm that this compound rapidly attenuated the excessive formation of protein aggregates in iPSC-derived neurons. Intriguingly, 4-PBA administration efficiently suppressed the percentages of apoptotic cell death in trisomy 21 and trisomy 13 neurons, indicating that this process might be a potential therapeutic target for the pathogenic features of DS.

4-PBA is a well-known small-molecule chaperone, classified as a hydrophobic compound. 4-PBA can slow or prevent protein misfolding and aggregation, likely via its chaperone effect on hydrophobic interaction^50^. Several reports have demonstrated that 4-PBA has neuroprotective effects in both in vitro and in vivo models of PD, AD, and HD. Treatment with 4-PBA decreases the aggregation of α-Syn or Htt protein and decreases tau phosphorylation in these cell culture^51,52^. Oral administration of 4-PBA to mouse models of neurodegenerative diseases was found to significantly ameliorate cognitive deficits^51,53^.

Recent research has provided increasing evidence of the relation between ER stress and autophagy^54^. Autophagic process is induced by unfolded or misfolded proteins that exceed the capacity of proteasome and is generally activated as a cytoprotective response for cells under severe ER stress^55^. Aneuploidy-associated stress is known to stimulate the accumulation of autophagosomal cargo such as protein aggregates within lysosomes^15^. Importantly, impaired autophagic flux, which is accompanied by elevated p62, has been observed in DS cells^56^. It was reported that 4-PBA alleviates ER stress and ER stress-associated autophagy^57,58^ by suppressing the AKT/TSC/mTOR signalling pathway^54^. Co-treatment with 3-MA, a macroautophagy inhibitor, reduced the suppressive effect of 4-PBA on protein aggregation, suggesting that 4-PBA can suppress protein aggregation mainly via its chaperone activity in the ER stress response and subsequent autophagy activation.

In addition to its function as a chemical chaperone, 4-PBA shows broad-spectrum inhibitory action towards class I and class II HDACs. This action can modify the chromatin structure, leading to dramatic alterations in the gene transcriptional network, including the increase or decrease in global gene expression^59^. Considering that trisomic fibroblasts in premature senescence show global transcriptional amplification^18^ and trisomic neurons exhibit accelerated protein synthesis, the HDAC inhibitory activity of 4-PBA may cause beneficial effects on gene transcription. However, in comparison with 4-PBA, we observed only limited effects of other HDAC inhibitors (valproic acid, class I/II; TMP-269 and tasquinimod, class IIa) on protein aggregation. Consistent with our results, structural modification of 4-PBA to remove its HDAC inhibitory activity has been reported to retain the ability to suppress neuronal cell death^60^. Taken together, the protective actions of 4-PBA on the survival of trisomic neurons might be based on synergistic effects of chaperone-like interactions and HDAC inhibition. Because 4-PBA has multifaceted effects, whether its suppressive action on neural cell death is directly attributable to the reduction in protein aggregation or to other unknown mechanisms is unclear. Further studies are warranted to elucidate the detailed mechanisms of 4-PBA in the cell survival of trisomic neurons. Nevertheless, 4-PBA has the advantage of being orally bioavailable, blood–brain barrier permeable, and approved by the US Food and Drug Administration (FDA) for the treatment of urea cycle disorders. Our observation that the prevention of protein aggregation was associated with improved cell viabilities of trisomic neurons leads us to propose a new therapeutic target for the treatment of DS.

## Methods

All in vitro and in vivo studies were approved by the Ethics Committee (approval number 13123-823), and Animal Research Committee (approval number 27-090-003), Graduate School of Medicine, Osaka University. All experiments were performed in accordance with the approved guidelines.

### Generation of human iPSCs

Dermal fibroblasts or peripheral blood mononuclear cells from two patients with trisomy 21 and one patient with trisomy 13 were obtained from patients who were admitted to Osaka University. Informed consent was obtained from each patient’s guardians in accordance with the Declaration of Helsinki. Two healthy dermal fibroblast cell lines were purchased from Lonza (Walkersville, MD, USA) (full details of the samples used in this study are provided in Table S1). Human iPSCs were generated using a Sendai virus (SeV) vector encoding tetracistronic factors (*OCT4*, *SOX2*, *KLF4*, and *c-MYC*) with or without the vector encoding *BRG1/BAF155*^19^ with the miR-302 target sequence (SeVp [KOSM302L]), as described previously^4,20^. iPSCs were cultured on mitomycin C (Sigma, St. Louis, MO, USA)-inactivated mouse embryonic fibroblast (MEF) feeder layers with human embryonic stem cell medium (hES medium) consisting of DMEM/F12 (Wako Pure Chemical Industries, Osaka, Japan), 20% KnockOut Serum Replacement (Gibco, Grand Island, NY, USA), 2 mM l-alanyl-l-glutamine (Wako), 1% MEM non-essential amino acid solution (Wako), 0.1 mM 2-mercaptoethanol (Sigma), and 5 ng/mL basic fibroblast growth factor (bFGF; Katayama Chemical, Osaka, Japan). After 2–3 weeks, individual colonies were isolated and expanded. During each passage of iPSC colonies, small interfering RNA L527 (GeneDesign, Osaka, Japan) mixed with Lipofectamine RNAi MAX (Life Technologies, Carlsbad, CA, USA) was applied, as described previously^20^, until complete removal of the SeV genome, which was confirmed by PCR. After 20 enzymatic passages using TryPLE Express (Thermo Fisher Scientific, Waltham, MA, USA), karyotyping analysis of Tri13 iPSCs revealed one clone containing normal chromosome 13 disomy cells. These spontaneously corrected (i.e. trisomy-rescued) disomy 13 cells were subcloned using the single-cell dilution method. One clone each of a trisomy-rescued disomy 13 iPSC (rDi13) line and an artificially corrected disomy 21 iPSC (cDi21) line, as previously described^29^, was used in the isogenic control experiments.

### Karyotype analysis and pluripotency assays

Karyotype analysis was performed by G-band analysis or Q-band analysis by Chromocenter (Tottori, Japan). Teratoma formation was assessed by injecting iPSCs (0.5–1.0 × 10^6^) into the testes of 8-week-old severe combined immunodeficient mice. Eight to ten weeks after iPSC transplantation, tumours were dissected and fixed with phosphate-buffered saline (PBS) containing 4% paraformaldehyde (Wako). Paraffin-embedded tissue was sectioned and stained with haematoxylin and eosin. For in vitro pluripotency assays, iPSCs were fixed in 4% paraformaldehyde/PBS for 15 min at room temperature and immunostained using the following primary antibodies: anti-OCT4 (1:200, Santa Cruz Biotechnology, Santa Cruz, CA, USA) and anti-SSEA4 (1:200, Millipore, Bedford, MA, USA), and DyLight-488-conjugated secondary antibodies (1:500, Thermo Fisher Scientific).

### Establishment of NPCs from iPSCs

Neural differentiation toward NPCs was performed as previously described^21,61^ with modifications. Briefly, EBs were cultured for 8 days in suspension with 2 µM dorsomorphin (Calbiochem, San Diego, CA, USA) and 10 µM SB431542 (SB; Tocris Bioscience, Ellisville, MO, USA) in hES medium deprived of bFGF, and then attached on Matrigel (BD Bioscience, Bedford, MA, USA)-coated dishes in neuronal medium (N2B27 medium) consisting of DMEM/F12, neurobasal medium (Gibco), 1X N2 supplement (Gibco), 1X B27 without vitamin A (Gibco), 1X Glutamax (Gibco), 2 mM l-alanyl-l-glutamine, 1% MEM non-essential amino acid solution, and 0.1 mM 2-mercaptoethanol, supplemented with 20 ng/mL bFGF for an additional 5 days. Neural rosettes that appeared in the centre of the attached EB colonies were carefully isolated using pulled glass pipettes from the surrounding flat cells, and then, small rosette clumps were seeded on Matrigel-coated dishes after gentle trituration and cultured in N2B27 medium supplemented with 20 ng/mL bFGF for an additional 5–7 days. During expansion, cells with non-rosette morphology were scratched off culture plates using either sterile glass pipettes or sterile plastic pipette tips, followed by the aspiration of undesirable material. Upon reaching approximately 90% confluence, cells were dissociated into single cells by incubation with TryPLE Express (Thermo Fisher Scientific) and re-plated on the Matrigel-coated culture dish in N2B27 medium plus bFGF. The culture medium was changed every other day, and cells were passaged every 2–3 days. Typically, NPCs between passages 5 and 10 were used for the analysis. For further differentiation, NPCs were dissociated with TryPLE Express, and 2 × 10^4^ cells/well were plated on Matrigel-coated 24-well plates with N2B27 medium supplemented with 10 ng/mL brain-derived neurotrophic factor (BDNF; R&D systems, Minneapolis, MN, USA) and 10 ng/mL glial cell line-derived neurotrophic factor (GDNF; R&D systems). The culture medium was changed every 3 days.

### Preparation of a *piggyBac* vector for expressing NGN2 and introduction into iPSCs

To robustly generate homogenous neurons from iPSCs, we introduced the NGN2 transcription factor into iPSCs using a *piggyBac* vector. This *piggyBac* vector, containing NGN2 under the control of the tetracycline operator rtTA and neomycin resistance gene, was generated from the PB-TA-ERN (Ef1a_rtTA_neo) vector backbone^28^. PB-TA-ERN was a gift from Knut Woltjen (Addgene plasmid # 80474). The generated vector was then co-transfected together with a pCMV-hyPBase vector (gift from Sanger Institute) encoding transposase into iPSCs using the Neon Transfection System (Life Technologies). After clone selection using neomycin, we established iPSCs carrying the tetracycline-inducible NGN2 construct (NGN2-iPSCs).

### Direct neural conversion from NGN2-iPSCs

NGN2-iPSCs were dissociated into single cells using TryPLE Express, and 1 × 10^4^ cells/cm^2^ were plated on Matrigel-coated 24-well plates (Thermo Fisher Scientific) or 96-well plates (Greiner Bio-One, Frickenhausen, Germany). NGN2-iPSCs were converted to cortical neurons (NGN2-neurons) after 14 days of culture in N2B27 medium supplemented with 10 ng/mL BDNF, 10 ng/mL GDNF, and 1 μg/mL doxycycline (Takara, Osaka, Japan). Half of the medium was changed every 2 days.

### Quantitative RT-PCR

RNA was isolated from NGN2-iPSCs and day 14 NGN2-neurons using the NucleoSpin RNA extraction kit (Macherey–Nagel, Oensingen, Switzerland). Reverse transcription was performed using the ReverTra Ace qPCR RT kit (Toyobo, Osaka, Japan). Quantitative RT-PCR was performed using THUNDERBIRD SYBR qPCR Mix (Toyobo). Gene expression was calculated relative to β-actin (ACTB) expression. All primer sequences are provided in Table S2.

### Immunocytochemistry

Immunocytochemistry was performed as described previously with some modifications^18^. Cells were fixed with 4% paraformaldehyde/PBS and permeabilized with 0.5% Triton X-100/PBS for 15 min. Then, cells were blocked with 5% foetal bovine serum/PBS for 30 min. For primary antibodies, cells were incubated at 4 °C for 16 h with the respective antibodies, such as anti-SOX1 (1:100, R&D Systems), anti-SOX2 (1:100, R&D Systems), anti-PAX6 (1:100, Stemgent, San Diego, CA, USA), anti-Nestin (1:200, eBioscience, San Diego, CA, USA), anti-TUJ1 (1:1,500, Covance, Berkeley, CA, USA), anti-MAP2 (1:1,500, Millipore), anti-TBR1 (1:200, Abcam, Cambridge, UK), anti-GFAP (1:1,500, DakoCytomation, Carpinteria, CA, USA), anti-p62 (1:100, Abcam), anti-NeuN (1:100, Millipore), anti-cleaved caspase 3 (1:800, Cell Signaling Technology, Beverly, MA, USA), anti-GRP78/BIP (Abcam), or anti-GADD153/CHOP (Santa Cruz Biotechnology). Cells were washed with PBS and then incubated for 60 min with secondary antibodies such as Alexa Fluor 488, 555, 594, or 647 (Thermo Fisher Scientific). Nuclei were counterstained with Hoechst 33342 (Dojindo, Kumamoto, Japan). The cytoplasm was stained with HCS CellMask Deep Red Stain (Thermo Fisher Scientific).

### Western blotting

Western blotting was performed as described previously with some modifications^18^. Briefly, cells were lysed with RIPA buffer (Wako) containing a protease inhibitor mixture (Roche Diagnostics, Basel, Switzerland). Equal amounts of protein (10 μg) were electrophoresed using 10% sodium dodecyl sulphate–polyacrylamide gels. Proteins were transferred to polyvinylidene difluoride membranes, washed with Tris-buffered saline containing 0.05% Triton X-100, and incubated with BlockingOne solution (Nacalai Tesque, Kyoto, Japan) for 60 min. Anti-p62 (1:1,000; MBL) and anti-ubiquitin (1:1,000; Santa Cruz Biotechnology) antibodies were used as the primary antibodies. Horseradish peroxidase-conjugated anti-rabbit or anti-mouse IgG antibodies (Promega, Madison, WI, USA) were used as the secondary antibodies. As a control, β-actin was detected with anti-β-actin pAb-HRP-DirecT (1:2000; MBL). Blots were visualized using Chemi-Lumi One L (Nacalai Tesque). Stained membranes were scanned with ImageQuant LAS 4000 (GE Healthcare, Menlo Park, California, USA). Quantification was performed using ImageJ software (https://imagej.nih.gov/ij/).

### Cell proliferation assay

The Click-iT EdU Alexa Fluor 488 Imaging Kit (Life Technologies) was used to detect proliferative cells according to the manufacturer’s instructions. Briefly, the thymidine analogue EdU was added to the medium of NGN2-neurons on day 7 or 14 and cultured for 6 h before fixation with 4% paraformaldehyde/PBS. Incorporated EdU was then detected by a click reaction—a copper-catalysed azide–alkyne cycloaddition—using a fluorescent Alexa Fluor dye containing a picolyl azide moiety. All images were taken by an IN Cell Analyzer 6000 (GE Healthcare), and the EdU-positive cell ratio was analysed using an IN Cell Developer Toolbox 1.9 (GE Healthcare).

### Protein synthesis assay

The Click-iT AHA Alexa Fluor 488 Protein Synthesis HCS Assay Kit (Life Technologies) was used to detect newly synthesized proteins according to the manufacturer’s instructions. Briefly, the amino acid analogue of methionine containing an azide moiety was added to the medium of NPCs and cultured for 2 h before fixation. Detection of the incorporated amino acid utilizes a chemoselective ligation or click reaction between an azide and alkyne, where the azido-modified protein is detected with an Alexa Fluor 488 alkyne. All images were taken by the IN Cell Analyzer 6000, and relative protein synthesis ability was analysed using the IN Cell Developer Toolbox 1.9.

### Aggregate detection assay

The PROTEOSTAT Aggresome Detection kit (Enzo Life Sciences, Farmingdale, NY, USA) was used to detect protein aggregates in NPCs or NGN2-neurons according to the manufacturer’s instructions. Briefly, protein aggregates in NPCs or neurons were evaluated using red fluorescent molecular rotor dye to specifically detect denatured protein cargo within aggresome and aggresome-like inclusion bodies in fixed and permeabilized cells. Nuclei were counterstained with Hoechst 33342. The cytoplasm was stained with HCS CellMask Deep Red Stain. For drug treatment experiments, cells were treated with vehicle, 4-PBA (100 µM), TMP-269 (0.1 µM; Selleck, Houston, TX, USA), tasquinimod (0.1 µM, Selleck), or valproic acid (10 µM; Wako) from days 0 to 14, or with 3-methyladenine (3-MA, 10 µM; Sigma) for 24 h prior to aggregation analysis. All images were taken by the IN Cell Analyzer 6000 and were analysed using the IN Cell Developer Toolbox 1.9. Nuclei and cells were defined by Hoechst 33342 and CellMask, and the relative intensities of aggregates per cell were evaluated.

### Proximity ligation assay (PLA)

Proximity ligation assay was performed to detect and quantify protein–protein interactions in situ. It is based on the principle that if two proteins physically interact, antibodies bound to each of the proteins are in such close proximity to each other that oligonucleotides conjugated to the antibodies can serve as guides to ligate additional oligonucleotides to form a circular template for DNA synthesis^62^. The resulting amplified DNA can then be detected using a fluorescent dye-labelled probe. PLA was performed according to the manufacturer’s instructions as below. Cultured cells, fixed with 4% paraformaldehyde/PBS, were treated with Duolink PLA blocking buffer (Sigma) for 1 h at room temperature. Then, cells were incubated with two primary antibodies against two proteins of interest for 16 h at 4 °C and then with Duolink PLA secondary antibodies conjugated with PLUS and MINUS oligonucleotides at 37 °C for 1 h. DNA ligation, amplification, and detection (Duolink Red) were performed at 37 °C for 2 h. After cells were counterstained with Hoechst 33342, they were observed under a fluorescent microscope. PLA signals (red dots) per cell were detected using the IN Cell Analyzer 6000. PLA signals per cell were analysed using the IN Cell Developer Toolbox 1.9. Antibodies against the following proteins were used: α-Syn (Abcam), Htt (Cell Signaling Technology), p62 (Abcam), and Parkin (Abcam).

### Cell survival assay

NGN2-iPSCs were dissociated into single cells, and 1 × 10^4^ cells/cm^2^ were plated on Matrigel-coated 96-well plates with neural medium containing 1 μg/mL doxycycline. Plating efficiencies were evaluated by collecting and counting the cells on day 1. Cells were fixed and stained on days 7 and 14, and surviving neurons stained with NeuN were quantified by the IN Cell Analyzer 6000, adjusted by plating efficiencies. To evaluate its prophylactic effect, 4-PBA (Sigma) was added when the medium was changed, from day 0 to day 14, and the number of surviving neurons was evaluated. All images were taken by the IN Cell Analyzer 6000 and were analysed using the IN Cell Developer Toolbox 1.9.

### Statistical analyses

All statistical analyses were performed using R version 2.14.0 (https://www.r-project.org) software. Comparisons were made by the two-tailed Student’s *t*-test. We evaluated multiple comparisons by one-way ANOVA with Tukey’s honestly significant difference test. *P* < 0.05 was considered significant. Data and graph bars are expressed as the mean ± standard error of measurement.

## Supplementary information

Supplementary Figure S1.

Supplementary Figure S2.

Supplementary Figure S3.

Supplementary Figure S4.

Supplementary Figure S5.

Supplementary Figure S6.

Supplementary Table S1.

Supplementary Table S2.
