# Supplementary material for: 4-Phenylbutyrate ameliorates apoptotic neural cell death in Down syndrome by reducing protein aggregates
Source: Sci Rep. 2020 Aug 20;10:14047. doi: 10.1038/s41598-020-70362-x (PMC7441064; doi:10.1038/s41598-020-70362-x)
Supplement: Supplementary file 1 — Supplementary Figure S1. [file 41598_2020_70362_MOESM1_ESM.pdf]

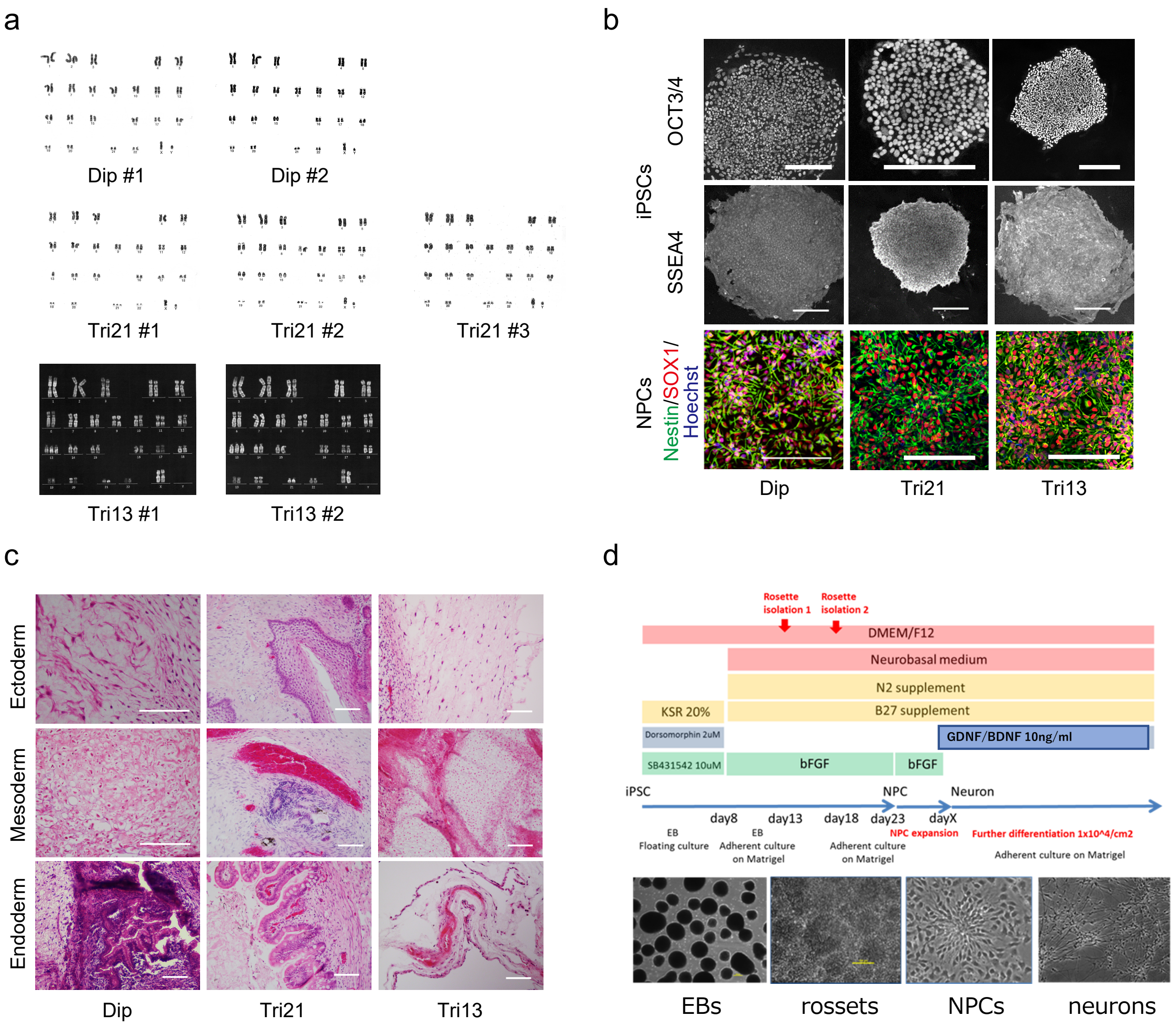

Figure S1

(a) Karyotype analysis of each iPSCs lines.

(b) Immunocytochemistry of each iPSCs using pluripotent markers OCT3/4 and SSEA4, and each NPCs using NPC markers SOX1 and Nestin. Scale bars = 200 μm.

(c) Representative images of a teratoma generated from respective iPSCs line. Scale bars = 200 μm.

(d) Schematic diagram showing the differentiation protocol of NPCs and cortical neurons.
