# Supplementary material for: 4-Phenylbutyrate ameliorates apoptotic neural cell death in Down syndrome by reducing protein aggregates
Source: Sci Rep. 2020 Aug 20;10:14047. doi: 10.1038/s41598-020-70362-x (PMC7441064; doi:10.1038/s41598-020-70362-x)
Supplement: Supplementary file 2 — Supplementary Figure S2. [file 41598_2020_70362_MOESM2_ESM.pdf]

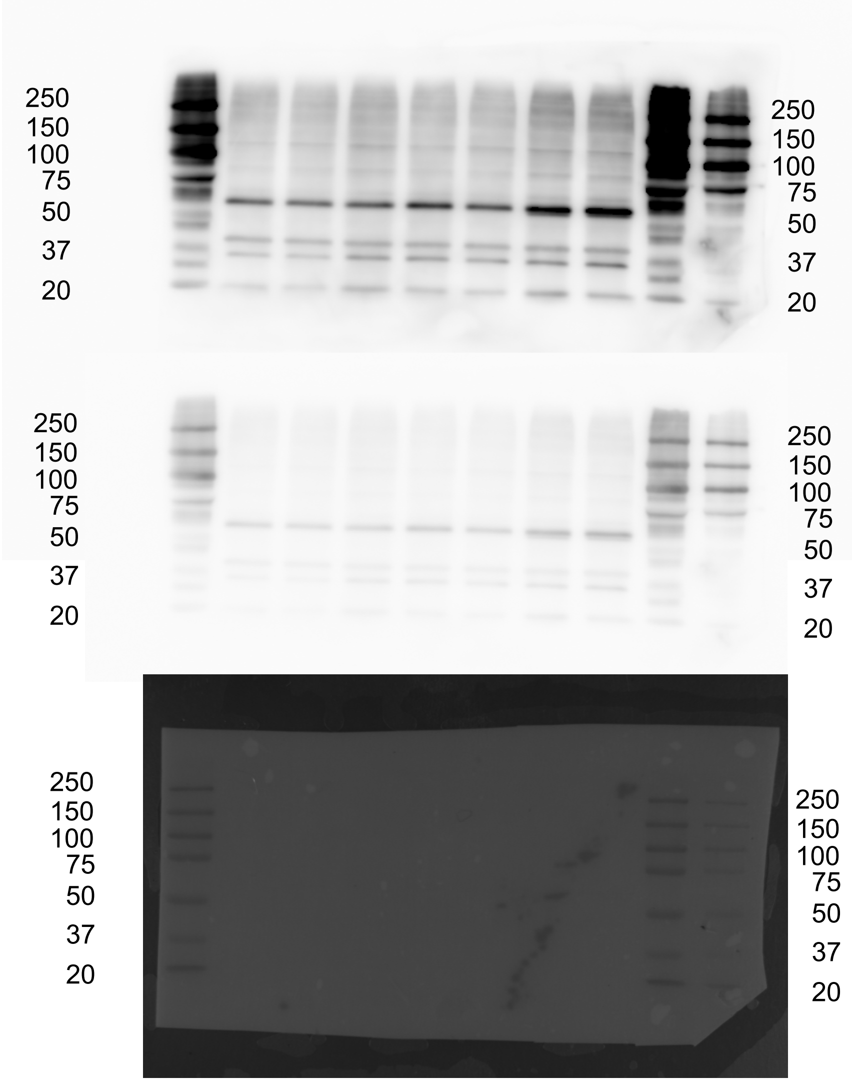

Figure S2

Full Blots of Figure 2d. Full immunoblot images of high-contrast (upper), low-contrast (middle) and size markers (lower) taken from the same membrane.
