# Supplementary material for: 4-Phenylbutyrate ameliorates apoptotic neural cell death in Down syndrome by reducing protein aggregates
Source: Sci Rep. 2020 Aug 20;10:14047. doi: 10.1038/s41598-020-70362-x (PMC7441064; doi:10.1038/s41598-020-70362-x)
Supplement: Supplementary file 3 — Supplementary Figure S3. [file 41598_2020_70362_MOESM3_ESM.pdf]

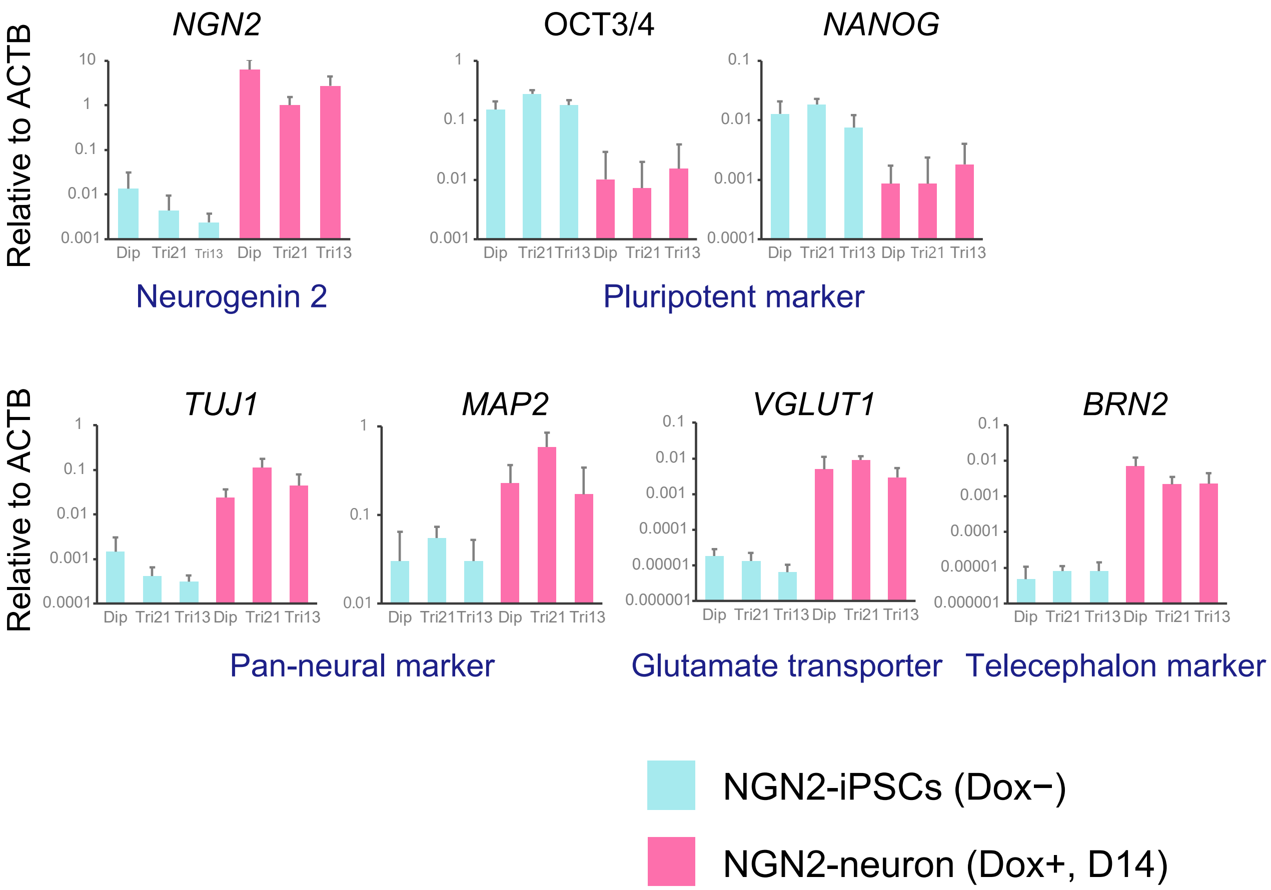

Figure S3

qRT-PCR analysis of the expression levels of the respective genes in NGN2-iPSCs and NGN2-neurons after 14 days of differentiation in diploid, trisomy 21, and trisomy 13 lines.

Data are presented as mean ± SEM. n = 3 per clone.
