# Supplementary material for: 4-Phenylbutyrate ameliorates apoptotic neural cell death in Down syndrome by reducing protein aggregates
Source: Sci Rep. 2020 Aug 20;10:14047. doi: 10.1038/s41598-020-70362-x (PMC7441064; doi:10.1038/s41598-020-70362-x)
Supplement: Supplementary file 4 — Supplementary Figure S4. [file 41598_2020_70362_MOESM4_ESM.pdf]

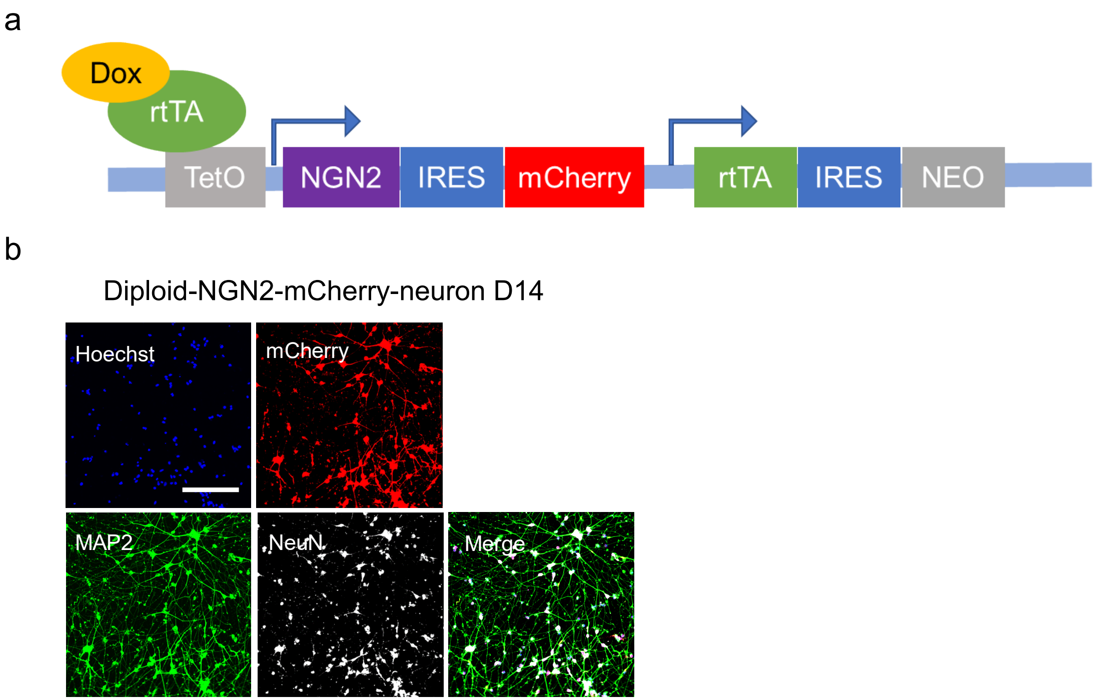

Figure S4

(a) Construct of NGN2-IRES-mCherry inducible *piggyBac* vector.

(b) Representative images showing immunocytochemistry of day 14 NGN2-IRES-mCherry neurons. Nearly 100% of cells expressed mCherry and were efficiently converted to neurons expressing the pan-neural markers MAP2 and NeuN. Scale bar = 200 µm.
