# Supplementary material for: 4-Phenylbutyrate ameliorates apoptotic neural cell death in Down syndrome by reducing protein aggregates
Source: Sci Rep. 2020 Aug 20;10:14047. doi: 10.1038/s41598-020-70362-x (PMC7441064; doi:10.1038/s41598-020-70362-x)
Supplement: Supplementary file 5 — Supplementary Figure S5. [file 41598_2020_70362_MOESM5_ESM.pdf]

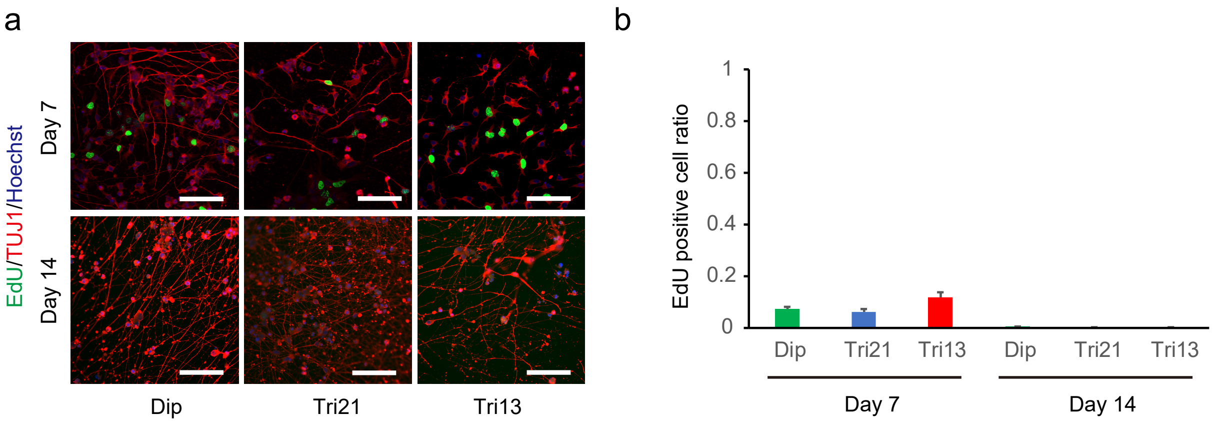

Figure S5

(a) Representative images showing immunocytochemical staining of EdU-positive cells (green) and TUJ1 (red) in diploid and trisomic NGN2-neurons on days 7 and 14. Hoechst 33342 counterstaining is blue. Scale bar = 200 µm.
(b) Quantification of the EdU-positive cell ratio. Data are presented as mean ± SEM. n = 4 per clone.
