# Supplementary material for: 4-Phenylbutyrate ameliorates apoptotic neural cell death in Down syndrome by reducing protein aggregates
Source: Sci Rep. 2020 Aug 20;10:14047. doi: 10.1038/s41598-020-70362-x (PMC7441064; doi:10.1038/s41598-020-70362-x)
Supplement: Supplementary file 6 — Supplementary Figure S6. [file 41598_2020_70362_MOESM6_ESM.pdf]

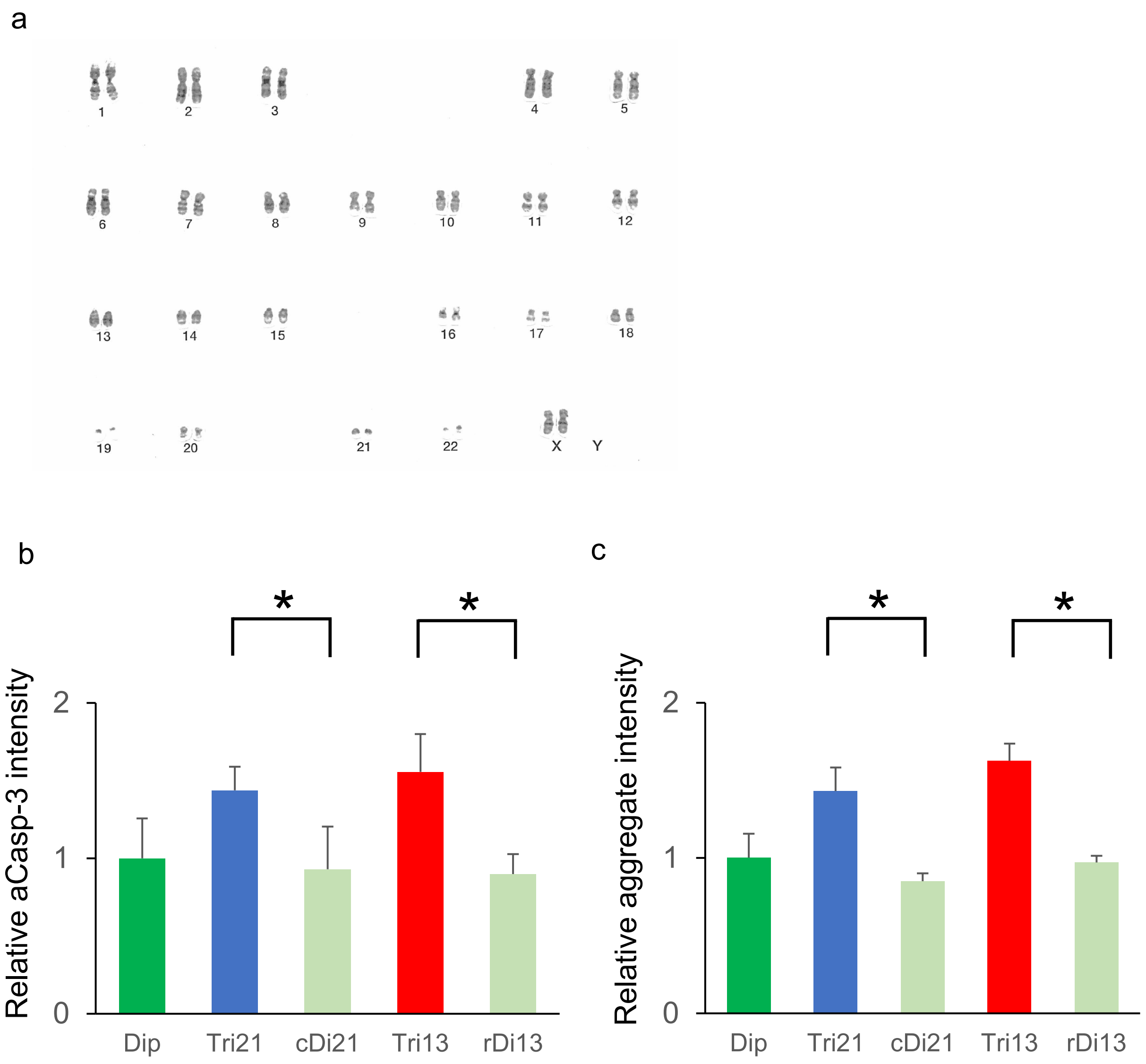

Figure S6

(a) Karyotype analysis of rDi13 iPSC. Karyotype analysis result of cDi21 is shown in Supplementary Figure S3 in our previous manuscript (Omori, 2017).

(b) Quantification of relative cleaved caspase 3 intensity in NGN2 neurons (day 14). *$P < 0.05$, $n = 3–9$ per clone.

(c) Relative aggregate intensity per cell in NGN2-neurons (day 14). *$P < 0.05$, $n = 3–6$ per clone.
