# Supplementary material for: 4-Phenylbutyrate ameliorates apoptotic neural cell death in Down syndrome by reducing protein aggregates
Source: Sci Rep. 2020 Aug 20;10:14047. doi: 10.1038/s41598-020-70362-x (PMC7441064; doi:10.1038/s41598-020-70362-x)
Supplement: Supplementary file 7 — Supplementary Table S1. [file 41598_2020_70362_MOESM7_ESM.docx]

**Table S1. Clinical features of trisomy syndrome patients in the present study**

| Patient | iPSC clone | iPSC source | Sex | Clinical manifestations | Age of death |
| --- | --- | --- | --- | --- | --- |
| Diploid-1 | Dip #1 | Dermal fibroblasts | M | - | - |
| Diploid-2 | Dip #2 | Dermal fibroblasts | M | - | - |
| Trisomy 21-1 | Tri21 #1 | Dermal fibroblasts | M | Tetralogy of Fallot, left renal hypoplasia, transient myeloproliferative disorder | 6y4m |
| Trisomy 21-2 | Tri21 #2 | Dermal fibroblasts | M | Patent ductus arteriosus, pulmonary hypertension, transient myeloproliferative disorder | 1m |
|  | Tri21 #3 | Peripheral blood mononuclear cells |  |  |  |
| Trisomy 13-1 | Tri13 #1 | Dermal fibroblasts | F | Hypoplastic left heart syndrome, cerebellar vermis deficit, cleft lip and palate | 2d |
|  | Tri13 #2 | Dermal fibroblasts |  |  |  |
