# Supplementary material for: 4-Phenylbutyrate ameliorates apoptotic neural cell death in Down syndrome by reducing protein aggregates
Source: Sci Rep. 2020 Aug 20;10:14047. doi: 10.1038/s41598-020-70362-x (PMC7441064; doi:10.1038/s41598-020-70362-x)
Supplement: Supplementary file 8 — Supplementary Table S2. [file 41598_2020_70362_MOESM8_ESM.docx]

**Table S2. Primers, Related to Experimental Procedures**

| **Gene** | **Forward primer (5’-3’)** | **Reverse primer (5’-3’)** |
| --- | --- | --- |
| *ACTB* | TCAAGATCATTGCTCCTCCTGAG | ACATCTGCTGGAAGGTGGACA |
| *NGN2* | GTGCAGCGCATCAAGAAGAC | CGGTGAGTGCCCAGATGTAG |
| *OCT3/4* | TGTACTCCTCGGTCCCTTTC | TCCAGGTTTTCTTTCCCTAGC |
| *NANOG* | CAG TCT GGA CAC TGG CTG AA | CTC GCT GAT TAG GCT CCA AC |
| *TUJ1* | CGGTGGTGGAGCCCTACAAC | AGGTGGTGACTCCGCTCAT |
| *MAP2* | AACCGAGGAAGCATTGATTG | TTCGTTGTGTCGTGTTCTCA |
| *vGLUT1* | CGCATCATGTCCACCACCAACGT | GAGTAGCCGACCACCAACAGCAG |
| *BRN2* | CGGCGGATCAAACTGGGATTT | TTGCGCTGCGATCTTGTCTAT |
